# Lentiviral-mediated gene correction of mucopolysaccharidosis type IIIA

**DOI:** 10.1186/1479-0556-5-1

**Published:** 2007-01-16

**Authors:** Donald S Anson, Chantelle McIntyre, Belinda Thomas, Rachel Koldej, Enzo Ranieri, Ainslie Roberts, Peter R Clements, Kylie Dunning, Sharon Byers

**Affiliations:** 1 Department of Genetic Medicine, Women's and Children's Hospital, Children, Youth and Women's Health Service, 72 King William Road, North Adelaide, SA 5006, Australia; 2 Department of Paediatrics, University of Adelaide, SA 5005, Australia; 3 Department of Biotechnology, Flinders University, GPO Box 2100, Adelaide, SA 5001, Australia; 4 School of Pharmacy & Medical Sciences, University of South Australia, GPO Box 2471, Adelaide, SA 5001, Australia; 5 Department of Respiratory and Sleep Medicine, Monash Medical Centre, VIC 3168, Australia; 6 Department of Obstetrics and Gynaecology, University of Adelaide, SA 5005, Australia

## Abstract

**Background:**

Mucopolysaccharidosis type IIIA (MPS IIIA) is the most common of the mucopolysaccharidoses. The disease is caused by a deficiency of the lysosomal enzyme sulphamidase and results in the storage of the glycosaminoglycan (GAG), heparan sulphate. MPS IIIA is characterised by widespread storage and urinary excretion of heparan sulphate, and a progressive and eventually profound neurological course. Gene therapy is one of the few avenues of treatment that hold promise of a sustainable treatment for this disorder.

**Methods:**

The murine sulphamidase gene cDNA was cloned into a lentiviral vector and high-titre virus produced. Human MPS IIIA fibroblast cultures were transduced with the sulphamidase vector and analysed using molecular, enzymatic and metabolic assays. High-titre virus was intravenously injected into six 5-week old MPS IIIA mice. Three of these mice were pre-treated with hyperosmotic mannitol. The weight of animals was monitored and GAG content in urine samples was analysed by polyacrylamide gel electrophoresis.

**Results:**

Transduction of cultured MPS IIIA fibroblasts with the sulphamidase gene corrected both the enzymatic and metabolic defects. Sulphamidase secreted by gene-corrected cells was able to cross correct untransduced MPS IIIA cells. Urinary GAG was found to be greatly reduced in samples from mice receiving the vector compared to untreated MPS IIIA controls. In addition, the weight of treated mice became progressively normalised over the 6-months post-treatment.

**Conclusion:**

Lentiviral vectors appear promising vehicles for the development of gene therapy for MPS IIIA.

## Background

The mucopolysaccharidoses (MPS) are a group of lysosomal storage disorders that arise from deficiencies in the catabolism of glycosaminoglycans (GAG) [1]. At present there are eleven known MPS, each resulting from the deficiency of a different lysosomal enzyme. Of the MPS, MPS IIIA (Sanfillipo A syndrome) is one of the most common, and as far as treatment goes, one of the most intractable, in that central nervous system (CNS) pathology is paramount [1]. Severely affected patients usually present by 2–3 years of age with a range of symptoms related to CNS pathology. These symptoms include delayed development, hyperactivity, aggressive behaviour, and sleep disturbances. Other symptoms include hirsutism and diarrhoea. The somatic manifestations of the disease, which include skeletal pathology, hepatosplenomegaly and joint stiffness, are generally milder and are more commonly found in older patients. MPS IIIA results from a genetically determined deficiency of sulphamidase, a lysosomal enzyme which normally catalyses the cleavage of N-linked sulphate from glucosamine residues at the non-reducing terminus of heparan sulphate. As this represents an obligatory step in the degradation of heparan sulphate, elevated levels of heparan sulphate fragments are found in tissues and in the urine. MPS IIIA also results in the secondary storage of GM2 and GM3 gangliosides in the CNS.

MPS IIIA represents a useful paradigm for therapies aimed at treating the widespread pathology which is found in many of the MPS. The availability of small (mouse) [2] and large (dog) [3,4] animal models of MPS IIIA provides a useful experimental resource for the preclinical development and testing of therapies.

The MPS IIIA mouse [2], the result of a spontaneous mutation, shows many of the progressive pathological features found in the human disease. By 6–7 months of age affected mice are noticeably less active, develop a scruffy appearance, hunched posture and abdominal distension, and lifespan is shortened. Affected animals show elevated levels of urinary GAG, which is predominantly heparan sulphate, greatly decreased sulphamidase activity in all tissues, normal or supranormal levels of other lysosomal enzymes and widely distributed storage. MPS IIIA mice are also significantly heavier than normals. The MPS IIIA mouse therefore provides an excellent model for the initial analysis of gene therapy strategies for the MPS in general, and MPS IIIA in particular.

Although intravenous enzyme replacement therapy has now been developed for a number of the MPS, it is obvious that this approach is not a viable option for treatment of CNS pathology due to its effective partitioning from the peripheral circulation by the blood-brain barrier [5]. Alternative therapies for MPS CNS pathology include small molecule therapies, aimed at preventing synthesis of storage material [6,7], and gene replacement therapy [7]. There is also accumulating evidence that the blood-brain barrier is not completely impermeable to lysosomal enzymes, and that high levels of enzyme in the peripheral circulation, delivered either by enzyme replacement therapy [8], or *via *gene therapy [9], result in delivery of significant amounts (i.e. high enough to affect pathology) of enzyme to the CNS.

Gene replacement therapy holds obvious potential for the treatment of the MPS [7,10,11], including MPS IIIA. We have previously demonstrated retroviral-mediated gene correction of cultured MPS IIIA fibroblasts [12]. However, retroviruses have serious limitations [13] that preclude their use in gene delivery to the CNS, and make them of limited utility in the transduction of any tissue *in vivo*. In order to further the development of gene therapy for MPS IIIA we have developed a lentiviral vector that expresses the murine sulphamidase gene and shown that it can be used to correct MPS IIIA cells *in vitro*. After intravenous administration of the vector to MPS IIIA mice urinary GAG and the weight of treated mice became progressively normalised over the 6-month period following vector administration.

## Materials and methods

### PCR

The primers used for amplification of the murine sulphamidase gene were msulatg (GGGCCCATCGATGCCACC *ATGCACTGCCCGGGACTGGCCTG*); msulbglr (*GAGGGTCGTAGATCTGGGGTGTCC*); msulbglf (*GGACACCCCAGATCTACGACCCTC*) and msultga (GGGCCCGAATTC *TCAGAGTTCATTGTGAAGCGGTC*). Sequences homologous to the murine sulphamidase gene sequence are shown in italics. The reaction conditions for all PCRs were 94°C, 30 seconds, 60°C, 20 seconds and 68°C, 1 minute for 20 cycles and used the Expand High Fidelity system (Roche). First strand cDNA from NIH3T3 cells (ATCC CRL-1658) was used as template. A 648 bp fragment corresponding to the 5' part of the murine sulphamidase cDNA sequence was amplified with the primers msulatg and msulbglr, and cloned as a *Cla*I/*Bg*lII fragment in pSP70 (Progen). A 872 bp fragment corresponding to the 3' part of the sequence was amplified with the primers msulbglf and msultga and cloned as an *Bgl*II/*Eco*RI fragment, again in pSP70.

### Lentiviral vector

The lentiviral vector used in this study was essentially the same as the pAF2Δ-SE vector [14] except that the SV40 early promoter was replaced with the murine phosphoglycerate kinase gene promoter from MSCVpac [15] to give pHIV-1pgkEYFP

### Virus production

The production and purification of the virus used in this work has been described elsewhere [16]. The virus was resuspended in 0.9% (w/v) NaCl and quantified by p24 ELISA (NEN-Dupont). Virus for *in vivo *administration was shown to be negative for replication competent virus [14].

### Cell culture, transduction, and enzymatic and metabolic analysis

Normal and MPS IIIA human skin fibroblasts were plated in 6-well plates and grown till confluent in DMEM/10% (v/v) FCS (2 mL per well). The medium was then aspirated and the cells fed with 1.5 mL of DMEM/10% (v/v) FCS containing 8 μg/mL polybrene. MPS IIIA cells were then transduced with vector for 24 hours. The medium was then exchanged for growth medium. For analysis, medium was exchanged for Ham's F12/10% (v/v) FCS, and after 4 hours the medium was exchanged again for Ham's F12/10% (v/v) FCS containing 10 μCi/ml ^35^SO_4_; a further 24 hours later the label was removed and the cells fed with DMEM/10% (v/v) FCS. To assess enzymatic cross-correction, labelled MPS IIIA cells were exposed to medium collected from lentivirus-transduced cells for 24 hours. For analysis of storage, cells were harvested 72 hours after labelling and cell lysates prepared by freeze/thaw in 20 mM Tris-HCl, pH 7.0, 500 mM NaCl. The cell lysates were then clarified by microcentrifugation (13,000 *g*, 5 minutes) and the supernatants assayed for sulphamidase [17] and β-hexosaminidase [18] activity, total protein and ^35^S cpm. The pellets resulting from the microcentrifugation of the freeze/thaw cell lysates were used to prepare genomic DNA using the Promega Wizard SV Genomic DNA kit.

### Real time PCR analysis

Vector sequences were detected in genomic DNA using a TaqMan assay for *gag *gene sequences present in the vector (Forward primer 5' AGCTAGAACGATTCGCAGTTGAT 3', reverse primer 5' CCAGTATTTGTCTACAGCCTTCTGA 3', probe 5' CCTGGCCTGTTAGAAAC 3' with FAM/NFQ reporter). Results were normalized using a single copy sequence in the transferrin gene (Forward primer 5' AAGCAGCCAAATTAGCATGTTGAC 3', reverse primer 5' GGTCTGATTCTCTGTTTAGCTGACA 3', probe 5' CTGGCCTGAGCTCCT 3' with FAM/NFQ reporter). The assays were run under standard conditions and using Applied biosystems TaqMan Universal PCR Master Mix. DNA from an A549 derived cell line, and containing a single copy of the lentiviral vector, was used to provide an absolute standard for copy number. Real time PCR was performed on an ABI 7300 cycler and analyzed using Sequence Detection Software v1.2.2 (Applied Biosystems). All samples were analyzed in triplicate.

### Treatment of MPS IIIA mice

The MPS IIIA mouse colony was originally established from mice provided by Dr. P. Stanley (Albert Einstein Institute College Medicine, New York). The mice were housed in the Women's and Children's Hospital Animal Care Facility where general maintenance was provided by trained animal care staff. MPS IIIA and normal mice were genotyped by PCR using previously established methods [19]. Six 5-week old male MPS IIIA mice were injected with 50 μg p24 equivalent of the lentiviral vector *via *injection into the tail vein. Three mice were pre-treated by intravenous injection of hyperosmotic mannitol (200 μl of 25% (w/v) mannitol in saline) 5 minutes prior to the administration of the vector in an attempt to achieve vector delivery to the CNS.

### Analysis of urinary GAG

Samples of mouse urine were incubated for one hour at 37°C with two volumes of 0.1% cetylpyridinium chloride in 0.054 M Na_3 _citrate (pH 4.8). Samples were centrifuged for 10 minutes at 3000 rpm and pellets were resuspended in 150 μL 2 M LiCl. Following addition of 800 μL absolute ethanol, samples were incubated at -20°C for one hour and then centrifuged for 10 minutes at 3000 rpm. Pellets were resuspended in 200 μL of water, lyophilised, and then resuspended in 20 μL water.

Purified glycosaminoglycan samples (0.2 μmol creatinine equivalents) were analysed on 40–50% linear gradient polyacrylamide gels as previously described [20].

### Statistical analysis

Except for weight data, results were analysed using one way ANOVA/SNK [21].

Mouse weights were analysed by comparison of non-linear trends for each group. Firstly, the relationship between body weight and age for each group was modelled using a cubic smoothing spline. Modelling using different non-linear trends for each group was then compared with modelling using the same non-linear trend for all groups and the log-likelihood values compared to determine significance. Where significance was found prediction intervals were calculated for the splines to determine the ages at which the groups were significantly different. In age intervals where predicted values for a group did not overlap the predicted interval for another group the treatment groups being compared were taken as being significantly different (p < 0.001).

## Results

### Isolation of the murine sulphamidase cDNA sequence and construction of lentiviral vectors transducing murine sulphamidase

**Figure 1:**
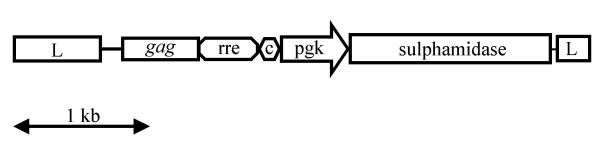
**Schematic representation of pHIV-1pgk*msulp *lentiviral vector**. Schematic of pHIV-1pgk*msulp *lentiviral vector showing pertinent elements. L, long terminal repeat, *gag*, *gag *gene sequence; rre, Rev-response element; c, central polypurine tract; pgk, murine phosphoglycerate kinase gene promoter; sulphamidase, murine sulphamidase cDNA sequence.

The murine sulphamidase cDNA sequence was PCR amplified in two parts as described in Materials and methods. The 5' primer used to amplify the 5' part of the sequence was designed to introduce a Kozak consensus sequence immediately prior to the initiation codon. DNA sequencing was used to confirm the absence of PCR induced errors. The two fragments were then joined *via *the common *Bgl*II site to generate a full length sequence. The full length sequence was then cloned into the pHIV-1pgk vector placing it under the transcriptional control of the pgk promoter (Fig. 1). The resulting construct was designated pHIV-1pgk*msulp*

### Correction of MPS IIIA skin fibroblasts, metabolic analysis

**Figure 2:**
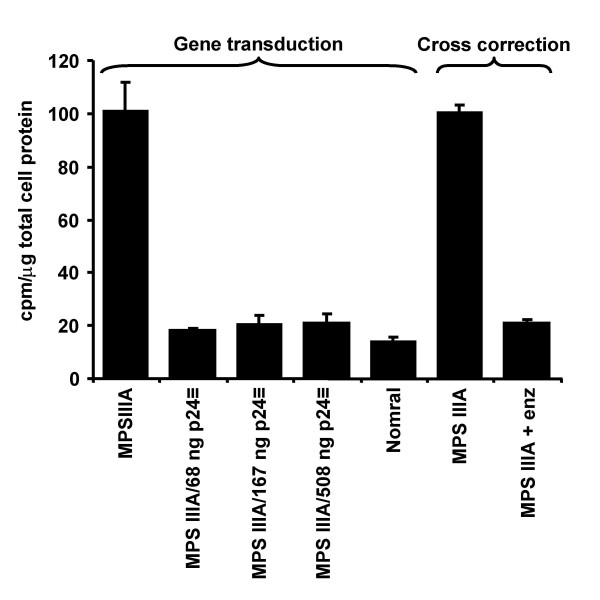
**Correction of MPS IIIA storage phenotype in vitro**. MPS IIIA cells were transduced with 68, 167 or 508 ng p24 equivalent of the pHIV-1pgk*msulp *vector and cell lysates assayed for incorporation of ^35^SO_4 _as described in Materials and methods (*Gene transduction*). Conditioned medium from the cells transduced with 508 ng p24 equivalent of vector was added to untransduced cells (*cross correction*), and again, cell lysates assayed for incorporation of ^35^SO_4_. Normal and untransduced MPS IIIA cells were used as controls.

MPS IIIA skin fibroblasts were transduced with 68, 167 or 508 ng p24 equivalent of the pHIV-1pgk*msulph *vector per well (6-well plate) as described in Materials and methods. Labelling with ^35^SO_4 _demonstrated that all vector transduced cells were metabolically normalised (Fig. 2), as were cells exposed to medium collected from cells transduced with 508 ng p24 equivalent of vector (Fig. 2). This medium contained 16 pmol/min/ml of sulphamidase activity. Conditioned medium from control MPS IIIA cells did not contain detectable sulphamidase activity. In all cases storage was significantly different from control MPS IIIA cells (P < 0.01) and not significantly different from control normal cells (P > 0.05).

### Correction of MPS IIIA skin fibroblasts, enzymatic analysis

**Figure 3:**
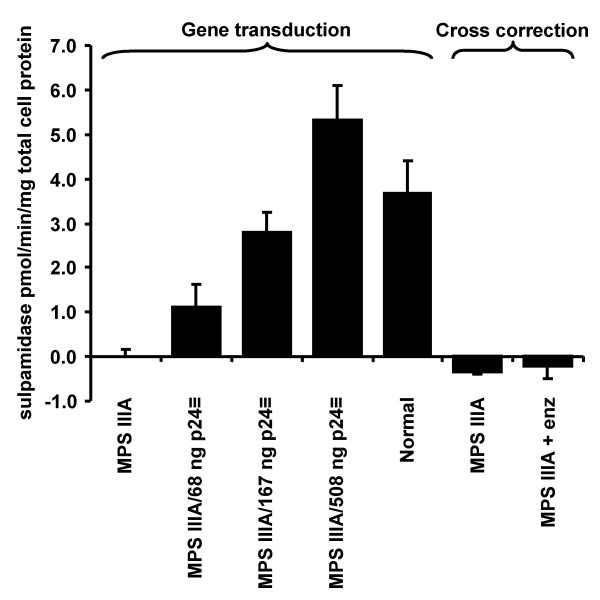
**Correction of MPS IIIA enzymatic phenotype in vitro**. MPS IIIA cells were transduced with 68, 167 or 508 ng p24 equivalent of the pHIV-1pgk*msulp *vector and cell lysates assayed for sulphamidase activity as described in Materials and methods (*Gene transduction*). Conditioned medium from the cells transduced with 508 ng p24 equivalent of vector was added to untransduced cells (*cross correction*), and again, cell lysates assayed for sulphamidase activity. Normal and untransduced MPS IIIA cells were used as controls.

Analysis of the levels of sulphamidase activity (Fig. 3) showed that the level of enzyme replacement increased with increasing amounts of vector added, with the two larger amounts effectively normalizing enzyme levels. In comparison to untransduced MPS IIIA cells, the increase in activity with the smallest amount of virus (68 ng p24) was insignificant, while the increase in sulphamidase activity with the two larger amounts of virus (167 and 508 ng p24) was significant (P < 0.01). The correction in enzyme activity in the cultures transduced with 167 ng p24 of vector was not significantly different from the level found in normal cells (P > 0.05), while enzyme activity resulting from transduction with 508 ng p24 of vector was significantly higher (P < 0.05) than in the normal control cells. β-hexosaminidase was not significantly different in any of the samples (P > 0.05). In the cross-correction experiments, sulphamidase activity in cells exposed to conditioned medium from transduced cells was not detectable above background.

### Correction of MPS IIIA skin fibroblasts, real time PCR analysis

**Figure 4:**
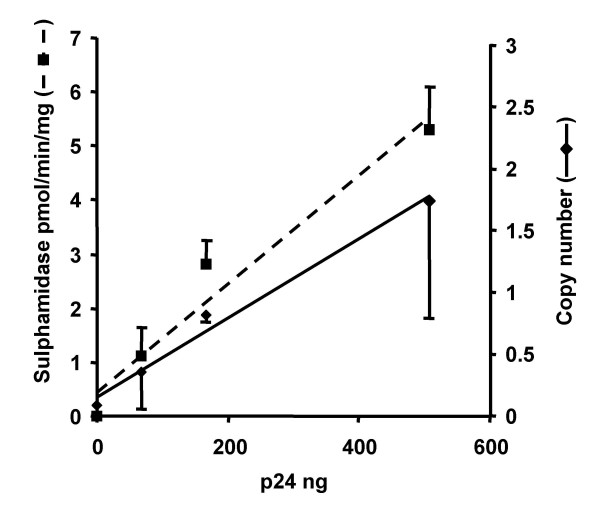
**Vector copy number in transduced MPS IIIA cells**. MPS IIIA cells were transduced with 68, 167 or 508 ng p24 equivalent of the pHIV-1pgk*msulp *vector and both vector copy number, and sulphamidase enzyme activity, were determined as described in Materials and methods. Both vector copy number and enzyme levels are proportional to vector dose.

Real time PCR analysis of DNA samples from transduced fibroblasts revealed the vector copy number to be proportional (R^2 ^= 0.99) to the dose of virus used (Fig. 4). The copy number varied from 0.3 copies/cell (68 ng p24 dose) to 1.7 copies/cell (508 ng p24 dose). No vector was detected in un-transduced cells or cells exposed to conditioned medium collected from transduced cells. Enzyme expression was proportional (R^2 ^= 0.90) to virus dose (Fig. 4), and hence also to vector copy number (R^2 ^= 0.95).

### *In vivo *administration of vector, analysis of urinary GAG and body weight

**Figure 5:**
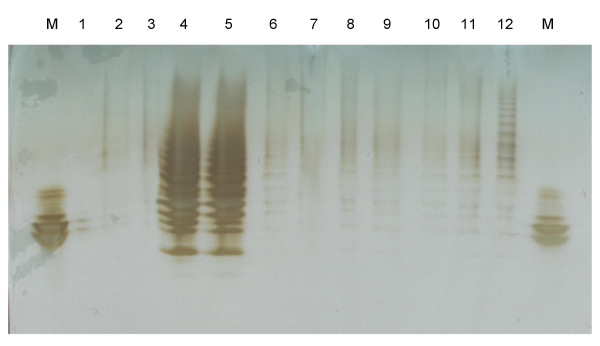
**Urine analysis**. Urine from selected mice was analysed by gradient PAGE as described in Materials and methods. Lane M, octasaccharide size standard; lane 1, empty; lane 2, normal; lane 3, normal; lane 4, MPS IIIA; lane 5, MPS IIIA; lane 6, treated #2, 54 days post-treatment; lane 7, treated #2, 122 days post-treatment; lane 8, treated #3, 67 days post-treatment; lane 9, treated #7, 61 days post-treatment; lane 10, treated #7, 196 days post-treatment; lane 11, treated #94, 106 days post-treatment; lane 12, treated #98, 98 days post-treatment.

Six 5 week old MPS IIIA mice (animal #s 2, 3, 7, 94, 98 and 99) were intravenously injected with vector as described in Materials and methods. Three of these mice (#s 2, 3 and 7) also received hyperosmotic mannitol immediately prior to administration of the vector. At various times post treatment urine was collected, GAG purified by CPC precipitation and analysed by gradient-PAGE as described in Materials and methods. The results of this analysis show that there is a large and consistent reduction in urinary GAG to normal, or near normal levels, in the treated animals (Fig 5). Comparison of samples taken from individual animals at different time points suggest that the reduction in urinary GAG becomes more marked after a longer period of treatment (e.g. animal 2, 122 days versus 54 days after treatment).

**Figure 6:**
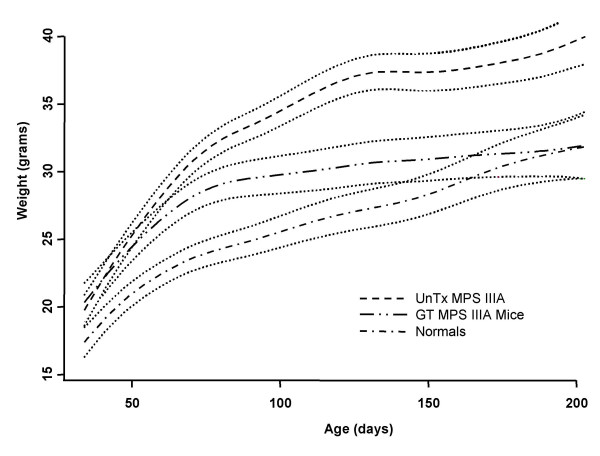
**Growth analysis**. Mouse weights were analysed by comparison of non-linear trends for each group as described in Materials and methods. The cubic smoothing spline and p < 0.001 prediction interval (dotted line) is shown for each group. ---, untreated MPS IIIA; -••-, treated MPS IIIA; -•-, normal.

At the time of treatment all animals showed the weight gain typical of MPS IIIA affected mice. However, over the 6-month treatment period their weight progressively trended towards normal and became closer to the normal range than to the range seen in untreated mice (Fig 6). No difference in growth was seen between the mice given hyperosmotic mannitol prior to treatment and those that were not (data not shown). Accordingly, the treated mice were analysed as one group. Statistical analysis showed that the weight of treated mice was significantly different from that of untreated mice after age 54 days (17–22 days post-treatment), and not significantly different from normal after age 166 days (18.5–19 weeks post-treatment).

## Discussion

Gene replacement therapy for the MPS has several potential advantages over enzyme replacement therapy, the current gold-standard for treatment where it is available. These include a reduced frequency of treatment, better efficacy and the prospect of being able to treat CNS disease by the introduction of gene vectors directly into the CNS. Until recently, the development of gene therapy for the MPS has foundered on the lack of suitable gene delivery vehicles. Generally, integrative vectors would seem to be preferable for inherited metabolic disorders such as the MPS as they confer genetic stability on the transduced gene and hence the potential for long-term effects. Because of this, retroviral vectors have long been the gene delivery vehicle of choice. However, vectors derived from oncogenic retroviral vectors are unable to transduce non-cycling cells [13], severely limiting their usefulness. For this reason we, and others, have developed lentiviral vectors [14,22-25]. These have the general positive attributes of retroviral vectors with the additional feature of being able to transduce non-cycling cells, meaning they have great utility for *in vivo *gene delivery. This has led to the use of lentiviral vectors in the development of gene therapy for a range of disorders, including the MPS [26-29].

In this study we have constructed a lentiviral vector and demonstrated proof of principle experiments *in vitro*. Human cells were used for these experiments simply for convenience; they were immediately available while cultures of control and MPS IIIA murine fibroblasts were not. Lentiviral-mediated gene delivery to human MPS IIIA skin fibroblasts resulted in correction of the metabolic and enzymatic defects exhibited by these cells, even at the lowest dose of virus used. In addition, the complete correction of the metabolic defect in cultured MPS IIIA cells with the lowest copy number of vector, and the fact that medium secreted from gene corrected cells was able to cross-correct the metabolic defect in non-transduced cells, demonstrates the potential of gene therapy to affect multiple cells in addition to those directly transduced by vector. In the cross-correction experiment it was not possible to assess whether enzyme uptake was *via *the mannose-6-phosphate (M6P) receptor, as metabolic correction was seen when enzyme was added in the presence or absence of M6P (data not shown). In addition, sulphamidase activity was too low to be detected in all samples from the cross correction experiment. This, at least in part, reflects the relatively low sensitivity of the enzyme assay, and in part the relatively low levels of enzyme activity in the conditioned medium used (towards the lower level of activity that can be easily detected).

In the *in vitro *study the pgk promoter appears to be relatively weak, as the level of expression obtained with the vector was not greater than that found in normal cells, suggesting it may be useful to assess the level of expression from other promoters. However, the use of strong promoters must be developed with caution as they increase the risk of insertional mutagenesis *via *oncogene activation [30].

The development of a real time PCR assay for our vector, and control cell line containing a single copy of our vector, will prove useful in further studies, for example determination of vector copy number in tissues after *in vivo *administration. By careful selection of the vector sequences that the real time PCR detects we have made this assay generic so that it will detect all versions of our vector, whatever the transgene or promoter sequence the vector carries.

Vector was administered to MPS IIIA mice either with or in the absence of a hyperosmotic mannitol pre-treatment. In the studies presented in this paper these two sets of animals could not be distinguished, therefore, all animals were grouped together for analysis. Administration of the vector to MPS IIIA mice resulted in partial normalisation of urinary GAG as evidenced by gradient PAGE analysis, giving an early indication of *in vivo *efficacy. The power of the gradient PAGE system [20] is that it allows the specific assessment of the small to large size free GAG molecules (i.e. four to thirty saccharide residues in length) typical of lysosomal storage material, rather than small free GAG (di- to tetra-saccharides) which can be analysed by mass spectrometry, or the total (free and conjugated) GAG measured by analysis of uronic acid.

The weight of the treated animals was also progressively normalised, suggesting that the treatment is having a widespread effect on the disease pathology even though enzyme activity could not be detected in blood samples from treated mice (data not shown). Further analysis of these animals is ongoing and will be published elsewhere.

In conclusion, lentiviral vectors appear to be promising reagents for the development of effective therapy for MPS IIIA. Future work will involve *in vivo *delivery of the vector to somatic and CNS cells and detailed analysis of the disease phenotype in treated animals.
